# Comparison between the standard and a new alternative format of the Summary-of-Findings tables in Cochrane review users: study protocol for a randomized controlled trial

**DOI:** 10.1186/s13063-015-0649-6

**Published:** 2015-04-16

**Authors:** Alonso Carrasco-Labra, Romina Brignardello-Petersen, Nancy Santesso, Ignacio Neumann, Reem A Mustafa, Lawrence Mbuagbaw, Itziar Etxeandia Ikobaltzeta, Catherine De Stio, Lauren J McCullagh, Pablo Alonso-Coello, Joerg J Meerpohl, Per Olav Vandvik, Jan L Brozek, Elie A Akl, Patrick Bossuyt, Rachel Churchill, Claire Glenton, Sarah Rosenbaum, Peter Tugwell, Vivian Welch, Gordon Guyatt, Holger Schünemann

**Affiliations:** Department of Clinical Epidemiology & Biostatistics, McMaster University, Hamilton, ON Canada; Department of Oral and Maxillofacial Surgery, Faculty of Dentistry, Universidad de Chile, Santiago, Chile; Institute of Health Policy, Management and Evaluation, University of Toronto, Toronto, ON Canada; Evidence-Based Dentistry Unit, Faculty of Dentistry, Universidad de Chile, Santiago, Chile; Pontificia Universidad Católica de Chile, Santiago, Chile; Departments of Medicine/Nephrology and Biomedical & Health Informatics, University of Missouri, Kansas City, MO USA; OSTEBA, Basque Office for Health Technology Assessment, Ministry for Health, Basque Government, Donostia-San Sebastián, Spain; Department of Medicine, Hofstra North Shore LIJ School of Medicine, Manhasset, NY USA; Iberoamerican Cochrane Center, Biomedical Research Institute Sant Pau-CIBER of Epidemiology and Public Health (CIBERESP-IIB Sant Pau), Barcelona, Spain; German Cochrane Center, Medical Center - University of Freiburg, Freiburg, Germany; Department of Medicine, Innlandet Hospital Trust-division, Gjøvik, Norway; Department of Medicine, McMaster University, Hamilton, ON Canada; American University of Beirut, Beirut, Lebanon; Department of Clinical Epidemiology, Biostatistics and Bioinformatics, Medical Center, University of Amsterdam, Meibergdreef, Amsterdam, The Netherlands; Center for Academic Mental Health, School of Social and Community Medicine, University of Bristol, Bristol, UK; The Norwegian Branch of the Nordic Cochrane Center, Oslo, Norway; Norwegian Knowledge Center for the Health Services, Oslo, Norway; Department of Medicine, Faculty of Medicine, University of Ottawa, K1H 8 M5 Ottawa, ON Canada; Bruyère Research Institute, University of Ottawa, Ottawa, ON Canada

**Keywords:** Summary-of-Findings table, Systematic review, Knowledge translation, Evidence summary, GRADE approach

## Abstract

**Background:**

Systematic reviews represent one of the most important tools for knowledge translation but users often struggle with understanding and interpreting their results. GRADE Summary-of-Findings tables have been developed to display results of systematic reviews in a concise and transparent manner. The current format of the Summary-of-Findings tables for presenting risks and quality of evidence improves understanding and assists users with finding key information from the systematic review. However, it has been suggested that additional methods to present risks and display results in the Summary-of-Findings tables are needed.

**Methods/Design:**

We will conduct a non-inferiority parallel-armed randomized controlled trial to determine whether an alternative format to present risks and display Summary-of-Findings tables is not inferior compared to the current standard format. We will measure participant understanding, accessibility of the information, satisfaction, and preference for both formats. We will invite systematic review users to participate (that is clinicians, guideline developers, and researchers). The data collection process will be undertaken using the online 'Survey Monkey' system. For the primary outcome understanding, non-inferiority of the alternative format (Table A) to the current standard format (Table C) of Summary-of-Findings tables will be claimed if the upper limit of a 1-sided 95% confidence interval (for the difference of proportion of participants answering correctly a given question) excluded a difference in favor of the current format of more than 10%.

**Discussion:**

This study represents an effort to provide systematic reviewers with additional options to display review results using Summary-of-Findings tables. In this way, review authors will have a variety of methods to present risks and more flexibility to choose the most appropriate table features to display (that is optional columns, risks expressions, complementary methods to display continuous outcomes, and so on).

**Trials registration:**

NCT02022631 (21 December 2013)

## Background

Systematic reviews play a major role in informing decision-making for clinicians, policy-makers, guideline developers, and other stakeholders [1,2]; however, their results are not always easy to understand and interpret. Studies regarding the use and interpretation of evidence to inform health care decisions have shown that text-reported information about a treatment effect is inconsistently interpreted [3,4], and numerical results of risks may be difficult to understand, even for educated users [5]. We previously [6] conducted a systematic review to study the effects of using alternative statistical presentations for reporting risk and risk reduction on understanding, perception, persuasiveness and behavior of health professionals, policy-makers, and consumers. Participants better understood risks expressed as natural frequencies (that is 15 out of 100) compared to risks expressed as probabilities (that is 15%). Relative risk reductions (RRR) were equally understood compared to absolute risk reductions (ARR), but better understood than number needed to treat (NNT), in particular when baseline risks were not shown. However, RRR were perceived as (misleadingly) larger and more persuasive than both ARR and NNT. These findings highlight the importance of appropriate presentation of systematic review results on users’ understanding, and perception.

The 'Grading of Recommendations Assessment, Development and Evaluation' (GRADE) system [7-12] is a widely accepted approach for developing and presenting summaries of evidence for both systematic reviews and clinical guidelines in a standardized and transparent manner [13]. The aim of this approach is to summarize the most important results from a systematic review in absolute and relative terms, be explicit about patient-important benefits and harms, and report the quality of the evidence (also called 'confidence in the effect estimates') for each outcome and across outcomes, in such a way that clinicians, policy- makers, and consumers can use reviews’ results efficiently and reliably. To accomplish this goal, the GRADE approach proposes the use of 'Summary-of-Findings' (SoF) tables and evidence profiles [14].

SoF tables have undergone deliberate development. The current format and content of SoF tables are based on user-testing studies, stakeholder feedback, and two randomized controlled trials [15-17]. These studies showed that SoF tables (incorporated in systematic reviews) significantly improved understanding (93% versus 44% (*P* = 0.003)) and the ability to find critical information (68% versus 40% (*P* = 0.021)) compared to those without SoF tables that report results only in a narrative way [17]. In another randomized trial, it was found that formatting modification of GRADE evidence profiles can increase the comprehension of key findings ranging between 5 to 47% [18].

Since 2004, The Cochrane Collaboration has been intensively working on the implementation of SoF tables in their reviews. By 2014, more than 600 SoF tables had been published across review groups (Langendam et al: Improving GRADE evidence tables: A systematic survey of explanatory footnotes and judgments in Summary of Findings Tables and Evidence Profiles shows more guidance is needed, manuscript in preparation). However, it has been noticed that the current standard format may need to be adapted depending on specific review characteristics. For example, the current SoF table format does not provide specific guidance for scenarios such as the narrative reporting of results when a meta-analysis was not undertaken nor alternative formats of presenting continuous and dichotomous outcomes. The inclusion of alternative presentations of risks in SoF tables included in systematic reviews would allow authors to choose from a variety of scientifically tested items that can be used to fit into the users’ needs.

Considering the above, the aim of this trial will be to determine whether new alternative SoF table format is not inferior compared to current standard format. Inferiority will be assessed by: understanding; perceived accessibility; satisfaction; and preference by health care professionals, guideline developers, and researchers that use and/or develop systematic reviews.

## Methods

The following description of methods and analysis of this trial follows the latest guidance by the Consolidated Standards of Reporting Trials (CONSORT) group in its extension for reporting of non-inferiority and equivalence randomized trials [19] and the SPIRIT (Standard Protocol Items: Recommendations for Interventional Trials) statement [20]. The protocol of this study was registered in the clinicaltrials.gov database (NCT02022631).

### Overview of the design

**Figure 1:**
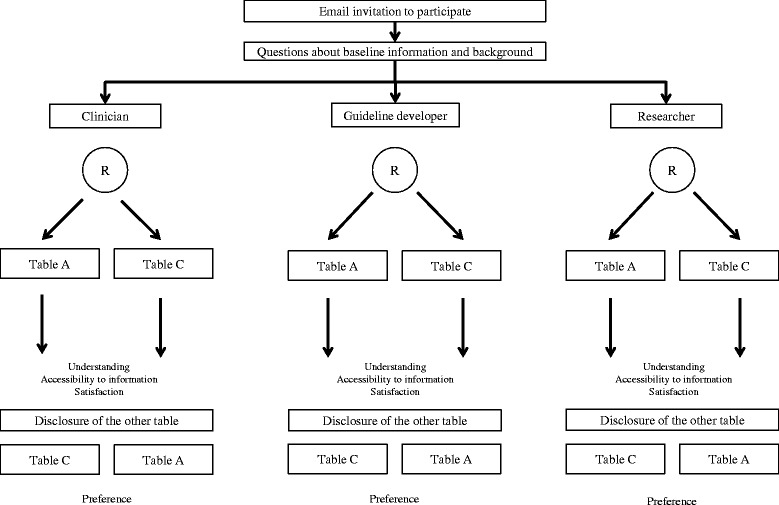
Study design and flow-chart.

We will conduct a parallel-armed, non-inferiority randomized trial comparing a new alternative format of SoF tables with current format. We will contact systematic review users by Email and ask them to fill a questionnaire developed using the 'Survey Monkey' online system. The data collection form will include questions about baseline information (demographic characteristics, background, familiarity with systematic reviews and the GRADE system, and so on). Then, participants will be randomly assigned to one of two SoF tables, either the one in the alternative format or the one with the current format (See Figure 1). Randomization will be stratified according to participants’ background (health professional, guideline developer, researcher). They will be asked to answer questions to determine understanding, accessibility of information, and satisfaction with the table format to which they were allocated. Finally, we will show them the other table format to which they were not initially allocated in order to test their preference for either one.

### Participants

#### Selection criteria

Participants will be eligible if they consider themselves as systematic review users. For the purpose of this trial, a user is defined as someone who has used Cochrane and/or non-Cochrane systematic reviews at least twice a year to answer clinical questions on a patient-health professional basis, inform the process of making recommendations for clinical practice guidelines, inform other types of evidence-based decision-making, or to use them for research purposes. We will include three target populations: (1) health professionals working in primary, secondary, or tertiary care, (2) clinical practice guidelines developers, and (3) researchers. We will classify as clinicians those who report at least 50% of total time dedicated to clinical practice. To be considered a guideline developer, participants should declare having participated in at least 1 clinical practice guideline during the last 2 years. Finally, participants who declare dedicating more than 70% of their time to conduct research (for example, methodologists, epidemiologists, statisticians, and so on) will be classified as researchers. Compared to previous trials testing SoF tables, this study includes similar populations [17,18].

#### Setting and recruitment

We will recruit participants from Europe, North America, South America, Africa and Australasia. We will contact people through various networks: Cochrane review groups and the networks of the co-authors who interact with guideline developers, researchers and systematic reviewers. Potentially eligible participants will receive a structured and standardized invitation with a link to access the 'Survey Monkey' questionnaire. Using this online system, we will determine whether the participants are eligible based on the selection criteria. All eligible participants will be provided with a brief explanation of the study and an online informed consent. We will also recruit participants at workshops, conferences and other research events. The Hamilton Health Sciences/Faculty of Health Sciences Research Ethics Board at McMaster University reviewed the protocol and approved it to be conducted without need of official approval, arguing that this trial is a quality improvement study with almost no risk to participants. Based on this assessment, participant consent was also waived by the Ethics Board.

### Intervention and comparison

**Figure 2:**
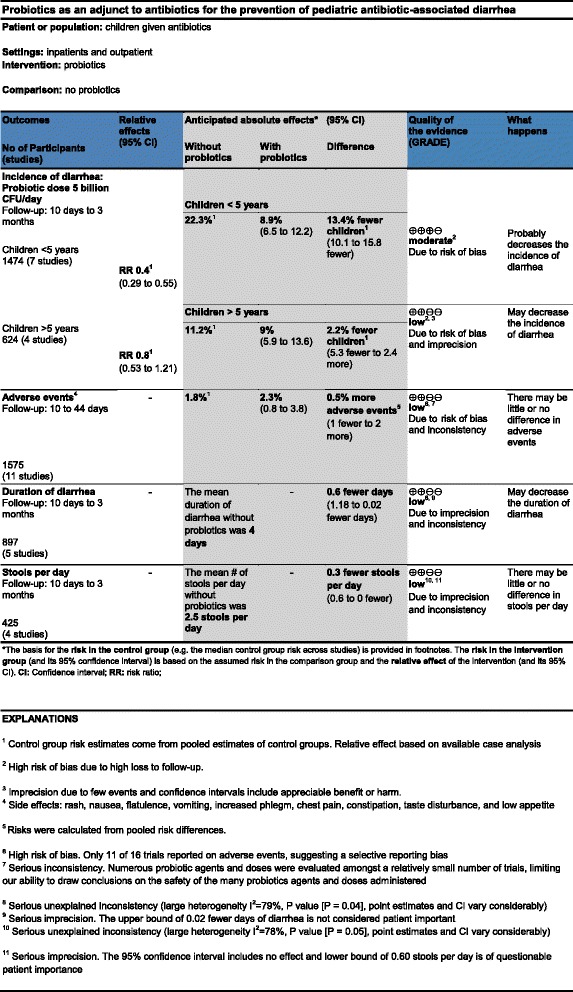
Alternative Summary-of-Findings (SoF) table format (Table A).

**Figure 3:**
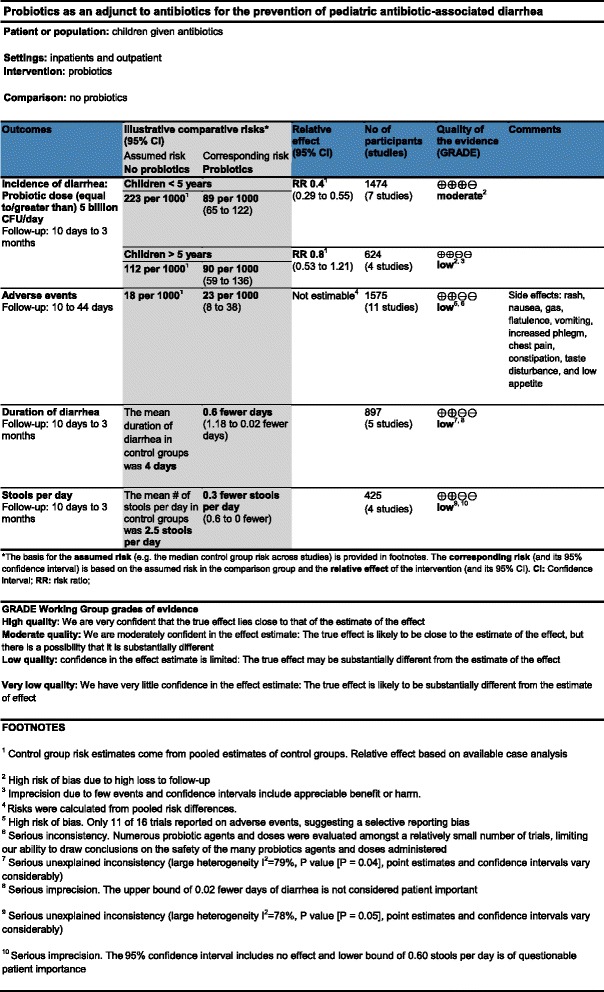
Current Summary-of-Findings (SoF) table format (Table C).

In this trial, one table displaying an alternative format of SoF will be tested against the current table format. In both SoF tables, the clinical question of the review in terms of patients, setting, intervention, comparator, outcomes, and the complementary information included as footnotes, will be the same. The only differences between the current and alternative SoF table formats will be the methods to either display the same data in a different way or to provide complementary data to the one shown in the current format (for example, risk difference). The SoF table in the current format that will be used in the control group is similar to the tables tested in previous trials on the same topic. With slight modifications, the SoF tables that will be used in this study are based on a real Cochrane systematic review conducted by Johnston *et al*. [21], titled *Probiotics for the prevention of pediatric antibiotic-associated diarrhea*. A comparison between the items included in the alternative and current SoF table formats is listed in Table 1. Figures 2 and 3 correspond to the alternative (Table A) and current formats (Table C) of SoF table formats respectively.

**Table 1:** **Comparison between items included in the current and alternative Summary-of-Findings (SoF) table formats**

	**Current formats (Table C)**	**Alternative formats (Table A)**
1	Inclusion of the number of participants and studies column	Exclusion of the number of participants and studies column. Information presented in the outcomes column
2	Quality of evidence presented with symbols and labeled as High, Moderate, Low, or Very low. Reasons for downgrading presented in the footnotes	Quality of evidence presented along with main reasons for downgrading in the same column (for example, Moderate due to imprecision)
3	'Footnotes' label	'Explanations' label
4	Baseline risk and corresponding risk expressed as natural frequencies	Baseline risk and corresponding risk expressed as percentages
5	No column presenting absolute risk reduction (risk difference) or mean difference	Inclusion of a column presenting absolute risk reduction (risk difference) or mean difference
6	Comments column included	Comments column deleted
7	No 'What happens' column^a^	'What happens' column included^a^
8	Description of the GRADE Working Group grades of evidence definitions below the table	No description of the GRADE Working Group grades of evidence definitions

^a^The 'What happens' column aims to summarize both the treatment effect and the quality of the evidence in one short narrative statement.

#### Randomization

Once the potentially eligible participants have completed the general information and background questionnaire, those who meet the selection criteria will be stratified according to self-reporting data as clinicians, guideline developers, or researchers and randomly allocated to one of the 2 SoF tables in a 1:1 ratio. To minimize missing data, the 'Survey Monkey' system will randomly assign participants to one of the SoF tables immediately after stratification, providing them with a link to access the questionnaires and tables.

#### Concealment of allocation

Since random allocation of participants to a SoF table is done automatically by the 'Survey Monkey' system in real time following an unknown algorithm, without a pre-specified sequence, it will be not known in advance to which group the next participant will be allocated. Therefore, in the trial allocation concealment is guaranteed.

#### Blinding

The data collection process will be conducted automatically by the 'Survey Monkey' web platform. The SoF table formats will be labeled as A, or C; thus, participants will be allocated to Table A or C without any other information about the nature of the tables. Participants will be blinded to whether the table they are exposed to was the one in the current or alternative format while the outcomes understanding, accessibility, and satisfaction, are assessed. The only exception will be the outcome preference, as the only way to measure it is comparing the table to which they are randomized with the table to which they were not allocated. Therefore, participant blinding will be broken only after they have fully assessed the SoF table to which they were initially randomized. Once the data collection process is completed, the database will be prepared for statistical analysis and interpretation in a blinded fashion. Finally, the labels will be revealed after the analysis is done.

### Outcomes

The outcome assessments will be defined and conducted as follow:

#### Primary outcome

##### Understanding

It is defined as the correct comprehension of key findings. We will frame seven multiple-choice questions about key concepts in the table with five response alternatives for each question and only one correct answer. We will compare the proportion of correct answers between groups per question. A 10% difference was defined as the minimal important difference between groups.

#### Secondary outcomes

##### Accessibility of information

This outcome considers 3 domains: (1) how easy it is to find critical information in the table; (2) how easy it is to understand the information, and (3) whether the information is presented in a way that is helpful for decision-making. These three domains will be measured by presenting participants with 3 statements for which they have to indicate the degree of agreement: 'It was easy to find the information about the effects'; 'It was easy to understand the information', and 'The information is presented in a way that would help me making a decision'. Agreement will be measured using a 7-point Likert-type scale (1 = I strongly disagree, 2 = I disagree, 3 = I somewhat disagree, 4 = Neither agree nor disagree, 5 = I somewhat agree, 6 = I agree, and 7 = I strongly agree). The outcome overall accessibility of information will be measured directly using a 5-point Likert scale (1 = Very inaccessible, 2 = Inaccessible, 3 = Neither inaccessible nor accessible, 4 = Accessible, 5 = Very accessible), asking the participant to consider the 3 above domains together. For all these measures, we will compare the means per group in each domain and overall.

##### Satisfaction

Measured at an item level, we will ask participants which formatting features satisfy them the most (for example, 'In Table A, we included a column called 'What happens'. The purpose of this column is to assist users on the interpretation of both review results and quality of the evidence. Do you think this column should be included as an available feature in future versions of SoF tables?'). It will be measured as a dichotomous outcome and we will compare proportions per group.

##### Preference

Participants will answer the question: Between alternative (Table A) and current format (Table C) of SoF table, 'which table do you prefer?' It will be measured using a 7-point Likert-type scale (1 = I strongly prefer Table A, 2 = I prefer Table A, 3 = I somewhat prefer Table A, 4 = Same preference for Table A or C, 5 = I somewhat prefer Table C, 6 = I prefer Table C, 7 = I strongly prefer Table C), and it will be treated as a continuous outcome.

The outcomes considered in this trials are similar to the ones measured in previous randomized controlled trials and other observational studies testing SoF tables [16-18]. Table 2 presents a summary of outcomes measures.

**Table 2:** **Overview of outcome measures**

**Outcome measures**	**Scale**	**Measure**	**Analysis method**
Primary			
Understanding	Dichotomous	% of participants with correct answers	Multiple logistic regression
Secondary			
Accessibility of information (at a domain level)	Ordinal (treated as continuous)	1-7 Likert scale	Multiple linear regression
Overall accessibility of information	Ordinal (treated as continuous)	1-5 Likert-type scale	Multiple linear regression
Satisfaction	Dichotomous	% of participants satisfied with an item	Chi-square test
Preference	Continuous	1-7 Likert scale	Multiple linear regression

### Sample size calculation

Sample size estimation was conducted using the software Stata/SE 10.1 (StataCorp., College Station, TX, USA) for Macintosh (Apple Inc., Cupertino, CA, USA). Based on the primary outcome, the null hypothesis was that the proportion of participants who answer the understanding questions correctly is lower in the alternative format table group compared to the current format table group, while the alternative hypothesis was that the proportion of participants correctly answering the questions in the alternative format table group is at least the same or higher compared to the current format table group (See Equation 1):

Null hypothesis

1 $$ {\mathrm{H}}_0:\ \mathrm{C}\mathrm{F}\ \mathrm{S}\mathrm{o}\mathrm{F}\ \mathrm{t}\mathrm{ables}\ \hbox{--}\ \mathrm{A}\mathrm{N}\mathrm{F}\ \mathrm{S}\mathrm{o}\mathrm{F}\ \mathrm{t}\mathrm{ables}\ {}^3\ \mathrm{N}\mathrm{IM}\left(\mathrm{C}\mathrm{F}\ \mathrm{is}\ \mathrm{superior}\ \mathrm{t}\mathrm{o}\ \mathrm{A}\mathrm{N}\mathrm{F}\right) $$

Alternative hypothesis

2 $$ {\mathrm{H}}_{\mathrm{a}:}\mathrm{C}\mathrm{F}\ \mathrm{S}\mathrm{o}\mathrm{F}\ \mathrm{t}\mathrm{ables}\ \hbox{--}\ \mathrm{A}\mathrm{N}\mathrm{F}\ \mathrm{S}\mathrm{o}\mathrm{F}\ \mathrm{t}\mathrm{ables}\ \le\ \mathrm{N}\mathrm{IM}\left(\mathrm{A}\mathrm{N}\mathrm{F}\ \mathrm{is}\ \mathrm{not}\ \mathrm{inferior}\ \mathrm{t}\mathrm{o}\ \mathrm{C}\mathrm{F}\right) $$

NIM: non-inferiority margin; CF: current format; ANF: alternative or narrative format

The proportion of participants correctly answering questions about understanding in similar randomized controlled trials that tested the current SoF table format was between 80 to 87% [17,18], and we expect, at least, the same percentage in the group of participants randomized to the alternative format of SoF table. A non-inferiority margin of 10% was set, and an allocation of 1:1 participants will be used. If there is truly no difference between the current and the alternative table format, then 280 participants are required to be 80% sure that the upper limit of a 1-sided 95% confidence interval (CI) will exclude a difference in favor of the current SoF table format of more than 10%. Assuming that 10% of participants would not complete the questionnaire, we aim to recruit a total of 308 participants. Linear and logistic regressions will be used as a method of analysis. According to Hsieh *et al*. [22], when more than one covariate is included in the model, a variance inflation factor should be added to the typical univariate analysis to obtain the required sample size. Equation 3 represents the variance inflation factor cited, where *b*_1_ represents the maximum likelihood estimate of *β*_1,_ var_p_(*b*_1_) corresponds to the variance of *b*_1_ that it is approximated from a one parameter model to a multivariate case when multiplied by 1/(1 - ρ^2^_1.23….p_), where ρ_1.23….p_ is the multiple correlation coefficient associating *X*_1_ with *X*_2_,…*X*_p_.

From unvariate to multivariate regression model sample size estimation:

3 $$ \mathrm{v}\mathrm{a}{\mathrm{r}}_{\mathrm{p}}\left({b}_1\right) = \mathrm{v}\mathrm{a}{\mathrm{r}}_1\left({\mathrm{b}}_1\right)/\left(1\hbox{-} {\uprho^2}_{1.23\dots .\mathrm{p}}\right) $$

Since this is a clinical trial in which participants will be allocated to the study arms randomly, it can be assumed that no correlation exists between being assigned to a particular arm and any of the predictors considered for analysis. Then, the value of ρ in the current study is zero, and the estimated sample size is the one described above.

### Statistical analysis

#### Descriptive and exploratory analysis

In both trials, descriptive analysis will include participants’ baseline characteristics and outcomes, means and standard deviations (SD) for continuous variables and proportions for categorical variables.

### Inferential analysis

The primary outcome (understanding) will be estimated as the proportion of participants correctly answering each question per group. Data regarding this outcome will be analyzed using a multiple logistic regression per question, where the main predictor will be the arm to which participants were allocated (dichotomous - 2 categories). Other potential predictors to include in the model are participant strata (nominal - 3 categories), years of experience (nominal - 5 categories), familiarity with the GRADE approach (dichotomous - 2 categories), and previous education in health research methodology or epidemiology (ordinal - 3 categories). To adjust for multiplicity, the *P*-value will be modified using the Bonferroni correction for 7 multiple comparisons (<0.0035, CIs will be constructed with corresponding 1-sided z score 2.7).

For the secondary outcome, accessibility of information, we will calculate means, SDs and 95% CIs per domain. Data regarding this outcome will be analyzed using multiple linear regression where the main pre.dictor will be the arm to which participants were allocated (dichotomous - 2 categories). Other potential predictors included in the model will be participant strata (nominal - 3 categories), years of experience (nominal - 5 categories), familiarity with the GRADE approach (dichotomous - 2 categories), and previous education in health research methodology or epidemiology (ordinal - 3 categories). To adjust for multiplicity, the *P*-value will be modified using the Bonferroni correction for 4 multiple comparisons (<0.0125).

The secondary outcome, satisfaction, will be measured at an item level. The proportion of participants satisfied with either item included in Table A or C will be calculated per group.

For the secondary outcome, preference, linear regressions will be used. Since this outcome is obtained from a direct comparison of the tables (blinding is disclosed), there will be no main predictor of interest. However, we will control for the order in which the tables were shown to the participants (dichotomous - 2 categories). Other potential predictors to include in the model will be participant strata (nominal - 3 categories), years of experience (nominal - 5 categories), familiarity with the GRADE approach (dichotomous - 2 categories), and previous education in health research methodology or epidemiology (ordinal - 3 categories).

#### Evaluation of the models

All the predictors presented along with the outcomes were selected because they have been considered potentially related to that particular outcome and could help to predict the observed variability. Harrell’s method [23] will be applied for variable reduction. First, the model will be run including only one key predictor variable. Then, in an iterative process each predictor will be included along with the key predictor cited above in a model. If in any of these steps a predictor changes the parameter estimate by more than 10%, it will be retained in the model. Finally, the definitive multivariable model will consist of the key predictor chosen and all the variables that changed the parameter estimate by more than 10%. The key predictor for the outcomes understanding, and accessibility of information will be the arm to which the participants were randomly allocated. For the outcome participants’ preference for a table, the order in which the tables were shown will be the key predictor.

#### Claiming of non-inferiority (CI approach)

For the primary outcome understanding, non-inferiority of the alternative (Table A) format to the current standard format (Table C) of SoF tables will be claimed if the upper limit of the CI (for the difference of proportion of participants answering correctly a given question, using Table C as reference) is lower than a non-inferiority margin of 10%. This approach is more informative as each question is related to a particular item under testing in the tables. Only the test for superiority will be applied to the secondary outcomes.

#### Dealing with dropouts and missing data

Since this trial will use an online system to allocate participants and collect baseline and outcome data, participants can leave the studies at any time. To minimize the possibility of dropouts and missing data, several strategies will be considered: (1) to reduce the level of burden to participants, only one link will be sent, which includes all the required questionnaires and material, (2) the random allocation to the studies arms will occur after collecting all the baseline characteristics, allowing the minimization of the likelihood of missing answers, (3) the questions will be set in mandatory response mode, so the participants cannot continue to the next form page until all the questions from the current page are answered, (4) the questions measuring outcomes will be designed as 'one-click questions', avoiding open-ended questions as much as possible, and (5) from the beginning of the data collection, randomization, and outcome assessment, a short process will be designed as participants would require no more than 15 to 20 minutes to respond.

Since the 'Survey Monkey' system allows setting each question as requiring an answer before moving to the next section, missing data for a couple of questions within a questionnaire is not possible. However, as in any type of research, participants can leave the study whenever they want. When this happens, all the answers before leaving will be registered in our database, and all the ones that were not answered will be classified as missing from that point in the questionnaire until the end. If a participant leaves the study before randomization, the participant will be excluded and thus we will not consider this as missing participant data. On the other hand, if the person is randomized and does not complete the questions, an available case analysis will be used (that is using the data available until the participant left the study). By the nature of the online system used to allocate participants, there is no possibility for participants to cross over to the other study arm.

## Discussion

This study represents an effort to provide systematic reviewers with additional options to display review results using SoF tables. Since the current format of SoF table has shown to improve understanding and facilitate the rapid retrieval of key findings, with an average of 90 seconds compared to the full text review [17], this time, we plan to conduct a non-inferiority trial with the aim to test an alternative format that can perform at least as effectively as the current one (comparison at a table level). In this way, review authors will have a variety of methods to present risks and more flexibility to choose the most appropriate table features to display (these being optional columns, risks expressions, complementary methods to display continuous outcomes, and so on).

The current trial has several strengths. First, it utilizes feedback and comments from a broad audience of GRADE users with different interests. This feedback informed a series of iterative user-testing rounds to examine the potential usefulness of several alternative formats for SoF tables. Second, it builds upon previous randomized controlled trials comparing the validated current standard format with the new alternative format proposed. Third, following methodological suggestions provided by Akl *et al*.[6] for further trials conducted in the same field, we designed a methodologically sound non-inferiority parallel-armed randomized controlled trial. Fourth, it will include participants from different backgrounds, settings (these being clinicians, guideline developers, and researchers), and different languages, which increases the generalizability of the findings. Fifth, this trial follows the recommendations for reporting provided by the CONSORT group in its extension for reporting of non-inferiority and equivalence randomized trials [19].

This study also has some limitations. First, since the data collection is conducted using an online system, there will be limited control over the environment in which the questionnaire is completed (that is whether it is completed by the same person that the link was sent to, whether the participant used additional information while answering the questions that measures the outcomes, and so on). Second, multiplicity and the corresponding adjustments may influence the precision of the CIs, a situation that may facilitate to find inconclusive results.

## Trials status

In participants’ recruitment phase.

## Abbreviations

ANF: alternative and narrative formats
ARR: absolute risk reduction
CF: current formats
CI: confidence interval
CONSORT: Consolidated Standards of Reporting Trials
GRADE: Grading of Recommendations Assessment, Development and Evaluation
NIM: non-inferiority margin
NNT: number needed to treat
RRR: relative risk reduction
SoF: Summary-of-Findings
SPIRIT: Standard Protocol Items: Recommendations for Interventional Trials
